# Inhibition of Histone Deacetylase Impacts Cancer Stem Cells and Induces Epithelial-Mesenchyme Transition of Head and Neck Cancer

**DOI:** 10.1371/journal.pone.0058672

**Published:** 2013-03-20

**Authors:** Fernanda S. Giudice, Decio S. Pinto, Jacques E. Nör, Cristiane H. Squarize, Rogerio M. Castilho

**Affiliations:** 1 Laboratory of Epithelial Biology, Department of Periodontics and Oral Medicine, University of Michigan School of Dentistry, Ann Arbor, Michigan, United States of America; 2 Department of Oral Pathology, School of Dentistry, University of São Paulo, São Paulo, Brazil; 3 Department of Cariology, Restorative Sciences and Endodontics, School of Dentistry, University of Michigan, Ann Arbor, Michigan, United States of America; Uppsala University, Sweden

## Abstract

The genome is organized and packed into the nucleus through interactions with core histone proteins. Emerging evidence suggests that tumors are highly responsive to epigenetic alterations that induce chromatin-based events and dynamically influence tumor behavior. We examined chromatin organization in head and neck squamous cell carcinoma (HNSCC) using acetylation levels of histone 3 as a marker of chromatin compaction. Compared to control oral keratinocytes, we found that HNSCC cells are hypoacetylated and that microenvironmental cues (e.g., microvasculature endothelial cells) induce tumor acetylation. Furthermore, we found that chemical inhibition of histone deacetylases (HDAC) reduces the number of cancer stem cells (CSC) and inhibits clonogenic sphere formation. Paradoxically, inhibition of HDAC also induced epithelial-mesenchymal transition (EMT) in HNSCC cells, accumulation of BMI-1, an oncogene associated with tumor aggressiveness, and expression of the vimentin mesenchymal marker. Importantly, we observed co-expression of vimentin and acetylated histone 3 at the invasion front of human HNSCC tumor tissues. Collectively, these findings suggest that environmental cues, such as endothelial cell-secreted factors, modulate tumor plasticity by limiting the population of CSC and inducing EMT. Therefore, inhibition of HDAC may constitute a novel strategy to disrupt the population of CSC in head and neck tumors to create a homogeneous population of cancer cells with biologically defined signatures and predictable behavior.

## Introduction

Among malignant head and neck tumors, head and neck squamous cell carcinoma (HNSCC) is the most common epithelial neoplasia and is one of the six most common malignancies worldwide [1]. HNSCC is characterized by lesions in the oral cavity, larynx, and pharynx. In spite of efforts to develop biomarkers for early detection and prognosis, the survival of HNSCC patients has not significantly improved [2]. The development of new therapies that improve the survival and quality of life of patients with HNSCC is urgently needed.

The initiation and progression of cancer is primarily controlled by genetic and epigenetic events that influence gene expression [3]. Epigenetic changes can regulate gene expression independently of genomic mutations. Epigenetic alternations are commonly observed upon DNA methylation and histone modification [4], [5]. Histones can be modified post-translationally through lysine acetylation and ubiquitination, serine phosphorylation, sumoylation, and methylation of lysines and arginines [6]. Histone acetyltransferases (HAT) catalyze the transfer of an acetyl group from acetyl-co-A to the e-amino site of lysine, resulting in chromatin decondensation. In contrast, histone deacetylases (HDAC) act on lysine residues to compact chromatin and suppress gene transcription [7], [8]. Interestingly, the effect of HDAC on chromatin organization is also associated with the regulation and maintenance of stem cell pluripotency in coordination with numerous signaling pathways [9], [10]. However, chromatin condensation is also associated with chemoresistance in tumors [11]–[14]. This phenotype is partially attributed to specialized cells that reactivate stem cell-like transcription programs [15]. These cancer stem cells (CSC) are characterized by a high proliferative rate, aggressive behavior, metastatic potential, and the ability to self-renew [16]–[25]. CSC are important therapeutic targets for cancer [26], and the clinical benefit of directly targeting CSC is under investigation. We wanted to determine whether interfering with chromatin condensation, known to play a key role in the maintenance of normal stem cells [9], [10], would influence tumor behavior and CSC content. We observed hypoacetylated chromatin in a panel of HNSCC-derived cell lines and identified a distinct population of CSC in these cells. These observations prompted us to ask whether chromatin acetylation dictates the biological behavior of tumors and whether pharmacological interference with HDAC alters CSC behavior. We found that inhibition of HDAC disrupts the accumulation of CSC and paradoxically induces tumor cells to undergo epithelial-mesenchymal transition (EMT).

## Materials and Methods

### Cell lines and culture conditions

We used HNSCC cell lines generated from the surgical removal of primary tumors localized in the tongue (HN6, HN13 and Cal 27), pharynx (HN30), larynx (Hep2) and derived from a tongue tumor that metastasized to lymph nodes (HN12) [27], [28]. Normal oral keratinocyte spontaneously immortalized cell line (NOK-SI) was previously established and kindly provided by Dr. Gutkind from the National Institute of Dental and Craniofacial Research (NIDCR/NIH) [29]. The NIH/3T3 normal fibroblast cell line was obtained from the American Type Culture Collection (ATCC - Manassas, VA, USA) and cultured using DMEM (Hyclone, Thermo Fisher Scientific, Waltham, MA, USA) supplemented with 10% bovine calf serum (Hyclone, Thermo Fisher Scientific, Waltham, MA, USA), 100 units/ml penicillin, 100 µg/ml streptomycin, and 250 ng/ml amphotericin B (Hyclone, Thermo Fisher Scientific, Waltham, MA, USA). Cells were maintained in a 5% CO2-humidified incubator. Primary human dermal microvascular endothelial cells (HDMEC; Lonza, Walkersville, MD, USA) were prepared in serum-free endothelial basal medium (Lonza, Walkersville, MD, USA).

### Western blotting

Tumor cells were lysed with cell lysis buffer containing protease inhibitors and briefly sonicated. Total protein was resolved on a 10–15% sodium dodecyl sulfate-polyacrylamide gel electrophoresis (SDS-PAGE) and transferred to an Immobilon-FL polyvinyl difluoride membrane (Millipore, Billerica, MA, USA). Membranes were blocked in 5% nonfat dry milk containing 0.1 M Tris (pH 7.5), 0.9% NaCl and 0.05% Tween-20 for 1 hour at room temperature. Membranes were incubated with Vimentin (clone V9, 1 500, Dako, Carpinteria, CA, USA), BMI-1 (1 500, Millipore, Billerica, MA, USA), Acetyl-Histone H3 Lys9 (1 1500, Cell Signaling, Danvers, MA, USA) or Acetyl-Histone H4 Lys 5, 8, 12 and 16 (1 2000, EMD Millipore, Billerica, MA, USA) primary antibodies at 4°C overnight. Membranes were then incubated with appropriate secondary antibodies conjugated to horseradish peroxidase (Santa Cruz Biotechnology, Sta. Cruz, CA, USA) for 2 hours at room temperature. The signal was developed using the ECL SuperSignal West Pico Substrate (Pierce Biotechnology, Rockford, IL, USA), and proteins were visualized using the UVP machine (BioSpectrum Imaging System). GAPDH served as a loading control (1∶20.000, Calbiochem, Gibbstown, NJ, USA).

### FACS of head and neck CSC

Head and neck cancer stem cell-like cells were identified by cell sorting for ALDH (aldehyde dehydrogenase) activity. The Aldefluor kit (StemCell Technologies, Durham, NC, USA) was used according to the manufacturer’s instructions to identify cells with high ALDH enzymatic activity. Briefly, HN6 and HN13 cells were treated with 300 nM Trichostatin A (Sigma-Aldrich Corp., St. Louis, MO, USA) for 24 hours and suspended with activated Aldefluor substrate (BODIPY-aminoacetate) or negative control (diethylaminobenzaldehyde, a specific ALDH inhibitor) for 45 minutes at 37°C. The samples were analyzed in the FACSDiVA Cell Sorter (BD Biosciences, Mountain View, CA, USA).

### Cell invasion assay

HN6 and HN13 cells (5×10^4^) were seeded in 24-well plates over a homogeneous thin layer of fibronectin (BD Biosciences, Bedford, MA, USA) in Millicell Cell Culture Inserts (Millipore, Billerica, MA, USA) that contained polycarbonate filter membranes with 8 µm-diameter pores. We determined that the optimal invasion time of HNSCC tumor cells was 8 hours after seeding, as evidenced by the substantial number of cells that invaded to the bottom of the polycarbonate filter membrane (∼60–70% of cells/total area) (Fig. S1). Tumor cells from the control group were maintained in DMEM supplemented with 10% FBS and 1% antibiotics, and the HDAC inhibitor group received 300 nm of Trichostatin A (TSA) diluted in media. The lower chamber contained DMEM supplemented with 20% FBS and 1% antibiotics. After plating, cells were incubated for 8 hours, based on optimal invasion time (Fig. S1), at 37°C in a 5% CO2-humidified incubator. Invasive cells in the lower chamber were stained with hematoxylin and eosin (H&E). Images were taken using a QImaging ExiAqua monochrome digital camera attached to a Nikon Eclipse 80i Microscope (Nikon, Melville, NY, USA) and visualized using QCapturePro software.

### IF-paraffin embedding, IF-cell lines, and antibodies

Human patient biopsies were embedded in paraffin, and 3-µm sections were used for immunofluorescence staining. Briefly, sections were incubated with primary antibodies overnight, washed with PBS, and incubated with a secondary antibody conjugated to either fluorescein (Jackson ImmunoResearch Labs 1:100) or rhodamine (Jackson Immuno Research Labs 1:100). Slides were mounted with DAPI-containing mounting media (Vector laboratories) and incubated at 4°C overnight with Vimentin (clone V9, 1:500, Dako, Carpinteria, CA, USA) and BMI-1 (1∶500, Millipore, Billerica, MA, USA) antibodies. For immunofluorescence analysis, cells were seeded on glass coverslips and fixed with methanol for 6 minutes at –20°C. Cells were treated with TSA (300 nM) or vehicle for 24 hours where indicated and stained for Ki-67 (1∶100, Dako, Carpinteria, CA, USA), Cytokeratin 14 (1∶1000, Covance, 155P), and rhodamine-phalloidin (1∶150, Cytoskeleton, Denver, CO, USA). Cells were co-stained with Hoechst 33342 (Sigma-Aldrich Corp., St. Louis, MO, USA) for visualization of DNA content. Images were taken using a QImaging ExiAqua monochrome digital camera attached to a Nikon Eclipse 80i Microscope (Nikon, Melville, NY, USA) and visualized with QCapturePro software.

### Sphere assay

To evaluate the ability of tumor cell lines to grow in suspension as spheres, HN6 and HN13 cells (10^3^) were cultured in ultra-low attachment plates (Corning; New York, NY, USA) for 5 days. Vehicle or TSA (Sigma-Aldrich Corp., St. Louis, MO, USA) was added to the culture media at a final concentration of 300 nM and closely monitored for 24 hours to determine the effect of hyper-acetylation in the maintenance of CSC spheres.

### Statistical analysis

Statistical analysis of invasion and proliferation rate of tumor cells was analyzed using an unpaired t-test. Sphere formation assay quantification was performed by one-way analysis of variance (ANOVA) followed by Tukey and Bonferroni multiple comparison tests. Assessment of cellular morphology and expression of vimentin were analyzed using GraphPad Prism 4.03 (GraphPad Software, San Diego, CA). Asterisks denote statistical significance (NS, P > 0.05; * P < 0.05; ** P < 0.01; and *** P < 0.001).

## Results and Discussion

### Chromatin acetylation and cellular behavior of HNSCC

**Figure 1:**
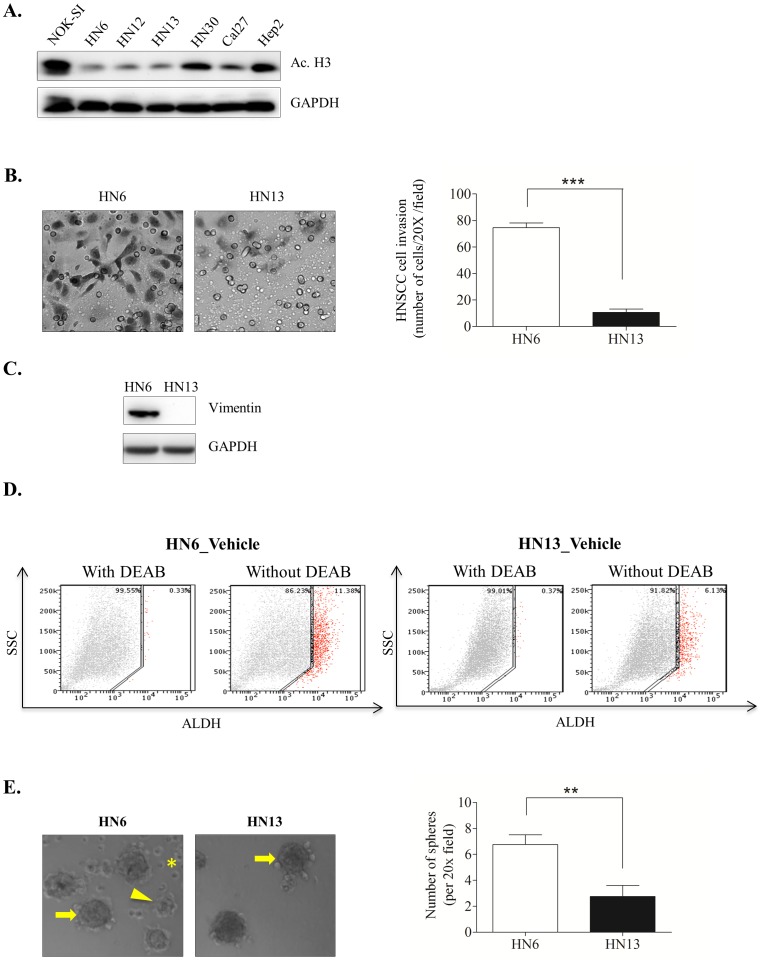
Hypoacetylation of tumor chromatin alone does not predict HNSCC behavior under normal culture conditions. (A) Western blot analysis showing global chromatin hypoacetylation of HNSCC, as evidenced by low expression levels of Acetyl-Histone H3 Lys9 (Ac.H3) compared to control normal oral keratinocytes (NOK-SI). (B) Representative images and bar graph of invasion assays. HN6 show a significant invasive capacity compared to HN13 (***p<0.001). Invasion was determined by counting HNSCC cells in the bottom of the membrane (see Materials and Methods for details) in multiple fields (20X). (C) Western blot analysis showing high expression levels of endogenous vimentin, a canonical marker of EMT, in HN6 cells but not in HN13 cells. (D) Evaluation of the CSC marker ALDH demonstrates that HN6 have a high number of ALDH+ cells, summing to more than 11% of the total tumor cell population. Approximately 6% of HN13 cells are ALDH+. (E) Representative example of holoclones (arrow), meroclones (arrowhead) and paraclones (asterisk) formed during the HN6 clonogenic assay. HN13 tumor cells primarily form undifferentiated holoclones (arrow). Quantification of spheres reveals increased ability of HN6 cell to generate spheres compared to HN13 (**p<0.01).

To investigate the role of chromatin remodeling in HNSCC behavior, we examined chromatin acetylation in a panel of 6 HNSCC cell lines. A spontaneously immortalized oral mucosa cell line (NOK-SI) was used as a control [29]. Histone proteins play structural and functional roles in all nuclear processes and undergo various modifications [30], including acetylation, methylation, phosphorylation, ubiquitination, SUMOylation, and poly-ADP-ribosylation to regulate chromatin structure and gene expression [31]. Acetylation of histone H3, commonly observed at Lys9, 14, 18, 23, 27, and 56, plays a role in gene activity [32], [33]. In particular, functional acetylation of histone 3 at Lys 9 has been extensively studied and is associated with histone deposition, chromatin assembly, and gene activation [34]–[36]. Tumor cells have varying acetylation levels of histone 3 at lysine 9, which is a marker of active genes [32], [33]. All HNSCC cell lines we analyzed displayed hypoacetylation of the chromatin compared to NOK-SI controls (Fig. 1A), suggesting HNSCC have condensed chromatin under basal culture conditions. Reduced acetylation levels of histone 3 at lysine 9 were also reported in tumors from lung and esophagus [37]–[39] and in 3D cultures of neuroblastoma cells and tumor spheroids derived from melanoma cells [40]–[42]. Alterations in chromatin organization play a role in many human diseases, including cancer [43], where they are considered a valuable prognostic marker [44].

We next examined the aggressiveness of HNSCC cells with low acetylation levels. HN6 tumor cells, which are sensitive to cisplatin (Almeida OA and Castilho RM, unpublished data), displayed enhanced invasiveness (Fig. 1B, ***p<0.001) and high expression of endogenous vimentin, an intermediate filament often found in aggressive malignant epithelial tumors that are undergoing epithelial-mesenchyme transition (EMT) [45]–[47] (Fig. 1C). Next, we sought to characterize the existence of a subpopulation of CSC known to corroborate to tumor initiation, growth, and metastasis [16]–[25] and are associated with the development of EMT [48]–[50]. Although several markers have been proposed to identify the CSC population within head and neck cancers, the activity levels of the enzyme aldehyde dehydrogenase (ALDH) is considered to be a highly selective marker for CSC and a stem cell biomarker for various normal and cancer stem cells [51]–[54]. Invasive HN6 have a presented a high number of ALDH-positive (ALDH+) cells, summing to more than which corresponded to over 11% of the total population (Fig. 1D_HN6). In contrast to HN6 cells, HN13 cells did not express vimentin, showed reduced invasiveness, and only 6% of their total cell population was comprised of ALDH+ cells Interestingly, HN13 tumor cells behaved differentially when compared to HN6 cells presenting a population of 6% of ALDH+ cells, absence of vimentin expression, and reduced invasive capacity (Fig. 1B, C, and D_HN13). In a clonogenic assay, HN6 and HN13 cells formed 3 different sphere patterns, identified as holoclones, meroclones and paraclones, that are directly associated with “stemness” Following, clonogenic assay of head and neck tumor cell lines displayed the formation of 3 different sphere patterns directly associated to the “stemness” behavior, the holoclones, meroclones and paraclones [55]–[57]. Holoclones are characterized by well-demarcated edges and possess the greatest growth potential, thereby likely to be composed of undifferentiated stem cells. Paraclones are characterized by fast growth, yet but limited cellular viability. Meroclones lie between the other two sphere patterns and are characterized by the two previous described CSC spheres patterns, by presenting colonies with wrinkled perimeters and enhanced cellular viability compared to paraclones [55]. We observed that invasive HN6 tumor cells generated more clones compared to HN13. Notably, sphere-forming cells from HN6 were a mix of holoclones (Fig. 1E_arrow), meroclones (Fig. 1E_arrow head) and paraclones (Fig. 1E_asterisk). However, HN13 spheres contained only large holoclones and no meroclones or paraclones (Fig. 1E_arrow), suggesting a homogeneous population of CSC.

Collectively, our results suggest that head and neck cancer cells consistently maintain hypoacetylated chromatin despite their aggressive behavior. Increased chromatin condensation is correlated to tumor resistance to chemotherapies [11]–[14], likely due to a disrupted influx of DNA repair molecules to the nucleus and impaired apoptosis [58]. In fact, chromatin decondensation is necessary for DNA repair [59]–[62], whereby members of the DNA repair machinery play a role in chromatin reorganization through large-scale chromatin unfolding [59], [63]. The presence of CSC, as detected by ALDH enzymatic activity, in human head and neck cancer cell lines is interesting and demonstrates the ability of tumor cells to maintain a heterogeneous population. Notably, we found that aggressive tumor cells are composed of a heterogeneous population of sphere-forming cells comprised of holoclones, meroclones, and paraclones and an overall large number of spheres (Fig. 1E, **p<0.01). The heterogeneous CSC pattern was not observed in indolent head and neck tumor cells. These findings suggest the coexistence of tumor cells with varying degrees of “stemness” that accounts for both their CSC population and invasiveness.

### Tumor microenvironment and HDAC inhibitor modulates chromatin acetylation and CSC content

**Figure 2:**
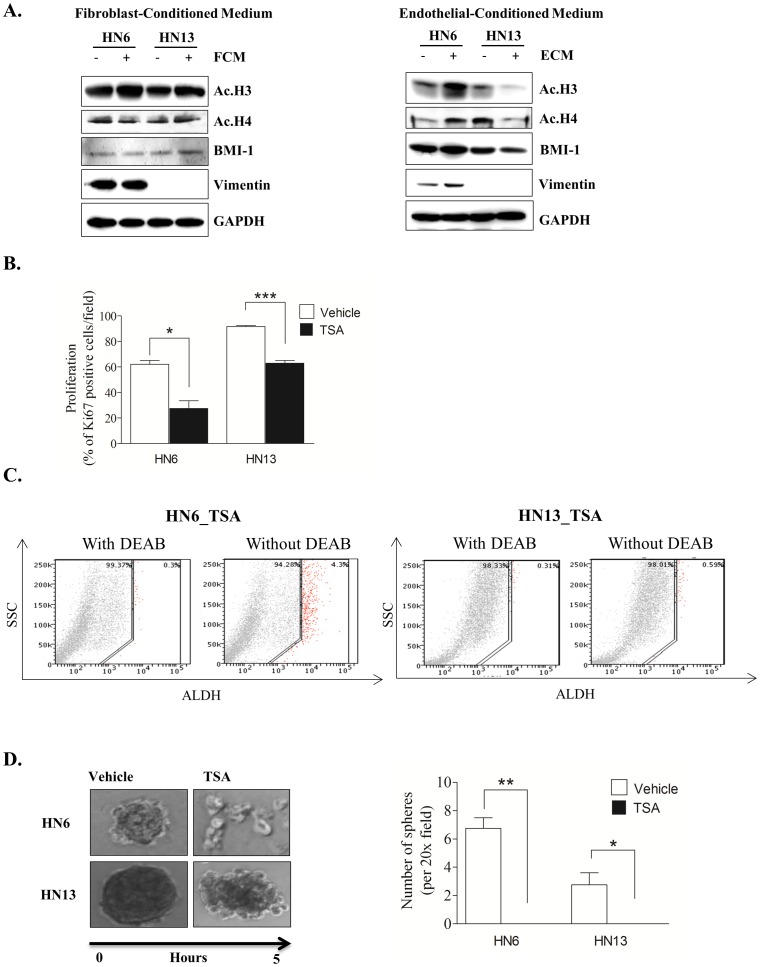
Acetylation of tumor chromatin is influenced by microenvironmental cues and dictates CSC fate. (A) Western blot analysis demonstrates that fibroblast-conditioned medium (FCM-left panel) does not modulate chromatin acetylation (Ac. H3 and Ac. H4), BMI-1 or vimentin levels in tumor cell lines. Endothelial-derived conditioned medium (ECM-right panel) influences tumor acetylation as depicted by elevated Ac. H3 and Ac. H4 levels in HN6 cells and ablation of Ac. H3 and Ac. H4 levels in HN13 cells. Note that along with Ac. H3 and Ac. H4, HN6 cells show minor increases in BMI-1 and vimentin levels. (B) Tumor proliferation assessed by Ki67 demonstrates reduced proliferation following administration of HDACi TSA (300 nM) for 24 hrs. (C) Administration of TSA (300 nM) for 24 hrs reduces the overall population of CSC in HN6 and HN13 cells, as evidenced by the presence of ALDH+ cells. (D) HDACi (TSA) disrupts tumor holoclones as depicted in representative images of tumor spheres and by quantification of spheres (HN6 **p<0.01, HN13 *<p<0.05).

Following our previous observations that tumor cells are found hypoacetylated, we decided to search for environmental cues that could influence chromatin acetylation during tumor invasion. We selected the arginine-rich histones H3 and H4 as markers for chromatin acetylation based on their ability to organize DNA within nucleosomes. Acetylated histones H3 and H4 release supercoiled DNA from nucleosomes to make genes more accessible [64]. Additionally, acetylation of histones H3 and H4 affects high-order chromatin structures and makes DNA binding sites accessible to trans-acting factors [65], [66]. We treated tumor cells with conditioned medium (CM) derived from fibroblasts and endothelial cells, two major components of the tumor microenvironment [67]–[69]. Although fibroblasts represent the major cellular component of the dermis and sub mucosa, CM from these cells failed to induce changes in acetylation of histones H3 (Ac. H3) and H4 (Ac. H4) (Fig. 2A_Fibroblast CM). However, CM from endothelial cells had a pronounced effect on the organization of tumor chromatin (Fig. 2A_Endothelial CM – Ac. H3 and Ac. H4). Interestingly, in response to CM from endothelial cells, HN6 cells displayed acetylated chromatin and increased expression of vimentin, which is mainly observed during EMT [45]–[47]. BMI-1, a member of the polycomb repressor complex 1 that is involved in chromatin remodeling and highly expressed in cancer cells [70]–[72], was upregulated in HN6 in response to endothelial CM (Fig. 2A_ Endothelial CM_HN6). Surprisingly, endothelial CM induced chromatin compaction in HN13 cells (Fig. 2A_ Endothelial CM_HN13) in a manner similar to the current two-step process of transcriptional repression mediated by the polycomb group family of genes (PcG). This process negatively influences DNA accessibility by transcriptional and remodeling factors, resulting in chromatin compaction (reviewed by Sparmann A, *et al*. [73]). Deacetylation of HN13 cells did not alter BMI-1 and vimentin expression (Fig. 2_Endothelial CM). Discrepancies between tumor behavior and chromatin response to environmental changes may be due to mutations in PcG family members that cause the aggressive behavior observed in HN6 tumors cells (Fig. 1B and C).

We next determined whether chromatin acetylation alone influences head and neck tumor behavior. We used the Trichostatin A (TSA) HDAC inhibitor to chemically induce chromatin acetylation. TSA selectively inhibits HDAC classes I and II, which possess epigenetic activity. Most recently, TSA has also been shown to inhibit non-histone transcriptional factors and co-regulators, including p53, STAT, and NFκB [74], [75]. After determining the optimal concentration of TSA (300 nM) capable of inducing head and neck cancer cell acetylation (Fig. S2) [76]–[82], we found that inhibition of HDACs directly impaired the proliferation of HNSCC cells (Fig. 2B, HN6 *p<0.05, HN13 ***p<0.001). We also observed an unexpected reduction in the fraction of CSC upon treatment with TSA (Fig. 2C_TSA), with a 7% reduction in ALDH+ cells isolated from HN6 and an approximate 6% reduction in ALDH+ cells from HN13 (Fig. 1D_Vehicle). As a functional assay, we evaluated the influence of TSA on the clonogenic formation of CSC spheres. Using ultra-low adhesion plates, we cultured tumor cells at low confluence for 5 days and observed until formation of well-defined spheres. Following sphere formation, TSA was administered, and the spheres were closely monitored. Surprisingly, induction of chromatin acetylation resulted in a rapid and progressive disruption of spheres (Fig. 2D, HN6 **p<0.01, HN13 *p<0.05). Disruption of the tumor spheres suggests that chromatin acetylation induced by HDAC inhibition disrupts the physiological requirements for CSC maintenance. Indeed, chromatin acetylation has long been known to induce cellular differentiation and restrict cellular transformation [83], [84]. Therefore, HDAC inhibitors (HDACi) may be a novel therapeutic strategy for impairing the deleterious effects of CSC. These results suggest a dynamic process in which head and neck cancer cells are highly susceptible to environmentally-driven epigenetic changes, supporting the notion that epigenetic targeting may be an effective and valuable approach for chemotherapy and chemoprevention of cancer [4]. HDAC inhibitors (HDACi) are drugs that target specific enzymes involved in the epigenetic regulation of gene expression and are potentially a new class of anticancer agents [4], [5], [7], [12], [85]. Although HDACi are successful in treating hematologic malignancies, their use in solid tumors remains controversial [86], [87].

### Chemically induced chromatin acetylation promotes EMT in HNSCC cells

**Figure 3:**
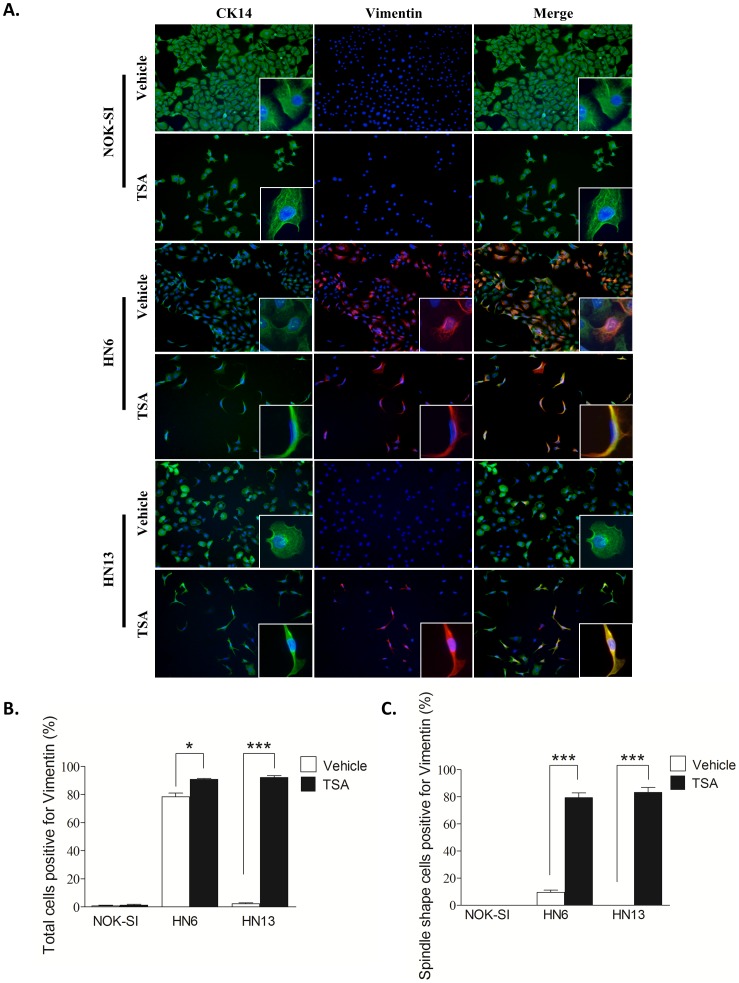
Chemically induced chromatin acetylation leads to activation of the EMT phenotype. Inhibition of histone deacetylase induces vimentin expression in HNSCC cells and acquisition of the spindle shaped morphology. (A) Representative examples of morphological changes and expression of the cytokeratin 14 (CK14) epithelial cell marker and the vimentin mesenchymal marker. Note that normal keratinocytes express CK14 in the presence of vehicle or TSA and cell morphology is continuously epithelioid (cobblestone or discoid appearance). Both HN6 and HN13 cells express CK14 and an epithelioid shape (A, vehicle). Following TSA treatment, HN6 and HN13 express vimentin and become spindle shaped. (B) Graphics represent the percentage of cells positive for vimentin following TSA or vehicle treatment. TSA-induced chromatin acetylation results in increased vimentin expression in HN6 and HN13 cells (*p<0.05 and ***p<0.001). (C) Graphic representing vimentin expression in spindle shaped cells (tumor cells with EMT-like morphology). HN6 and HN13 cells display significant increases in vimentin expression after TSA treatment (***p<0.001).

The effect of HDACi on CSC prompted us to determine whether administration of TSA would alter additional characteristics of head and neck cancer. Malignant tumors derived from epithelial cells (carcinomas) undergo an exquisite process known as EMT that precedes invasion and progression of cancer cells [46], [88]–[91]. EMT is characterized by loss of cell adhesion, increased motility, aggressive behavior, and acquisition of an elongated fibroblastoid morphology and expression of vimentin, a canonical marker of EMT [45]–[47]. Notably, aggressive HN6 cells had constitutive expression of vimentin (Fig. 1C) and a predominantly cobblestone appearance (Fig. 3A_Vehicle_HN6). Pharmacological inhibition of HDAC caused cells to rapidly alter their morphology into a spindle shape and to increase vimentin expression (Fig. 3A_ TSA_HN6). Furthermore, administration of TSA induced spindle morphology and vimentin expression in HN13 cells (Fig. 3A_ TSA_HN13) that do not typically express this intermediate filament (Fig. 1C_HN13_Vimentin). We did not observe TSA-induced morphological changes or vimentin expression in normal cells (Fig. 3A_NOK-SI), suggesting that hyperacetylation of chromatin differentially modulates normal and neoplastic cells. Although the total number of HN6 cells expressing vimentin only marginally increased in response to TSA (Fig. 3B_HN6, *p<0.05), tumor cells displaying fibroblastoid morphology were largely positive for vimentin (Fig. 3C_HN6, ***p<0.001). The combination of vimentin expression and fibroblastoid morphology was also observed in HN13 cells following chromatin acetylation (Fig. 3B and C_HN13 ***p<0.001). These results suggest a strong role for chromatin decondensation during acquisition of an EMT phenotype in HNSCC cells.

### Chromatin hyperacetylation enhances invasion and expression of BMI-1 in head and neck cancers

**Figure 4:**
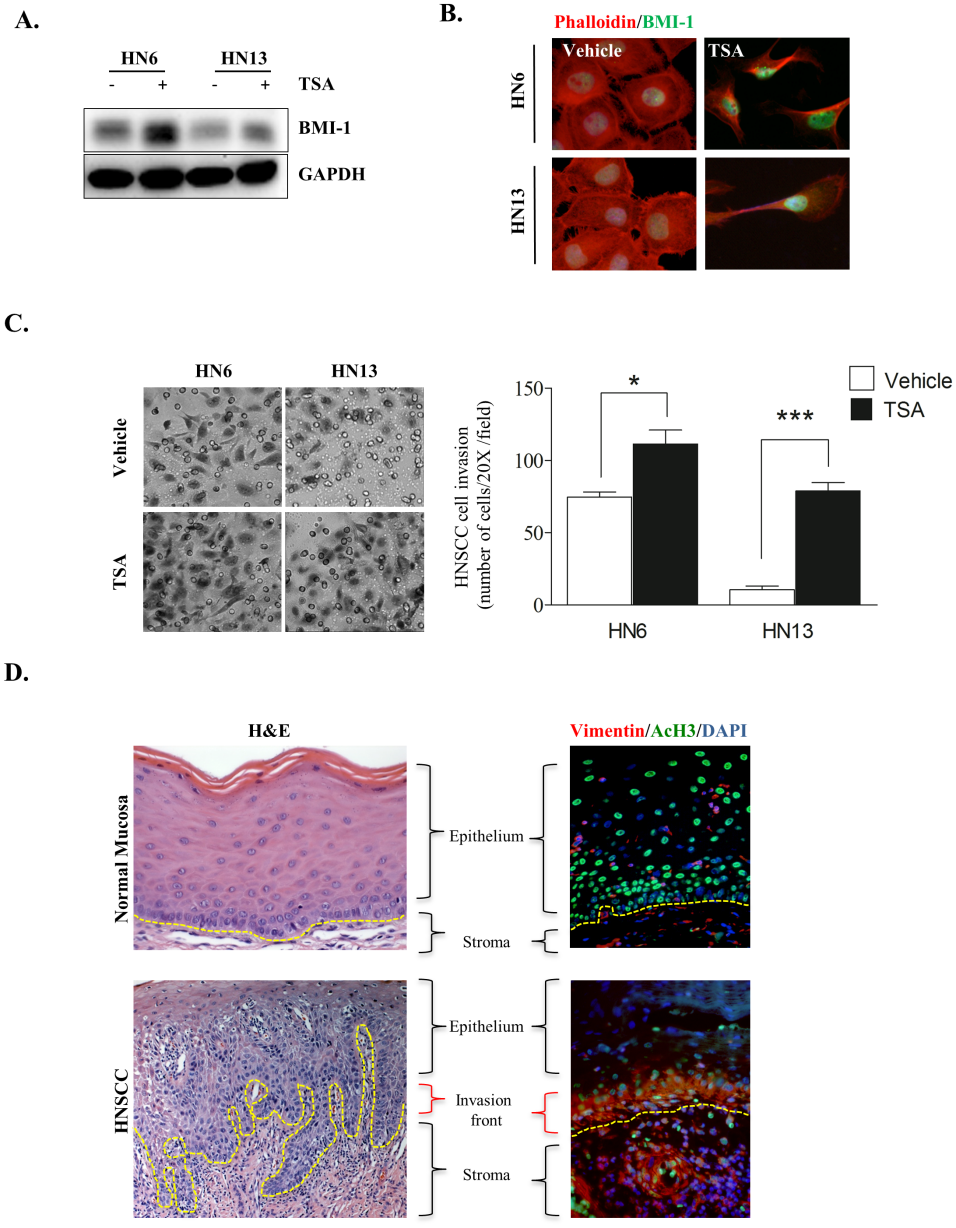
EMT phenotype induced by acetylation of tumor chromatin shows high BMI-1 levels and is observed in the invasion front of HNSCCs. (A) Western blot analysis depicting increased BMI-1 expression in HNSCC cells following TSA treatment. (B) Representative images of nuclear BMI-1 (FITC/green) and polarization of the F-actin filaments (TRITC/red) in tumor cells undergoing EMT after TSA treatment. (C) Representative examples of increased tumor cell invasion after treatment with HDAC inhibitors. Note the significant increase in invasiveness of HN6 and HN13 cells (* p<0.05 and *** p<0.001 respectively). (D) Representative examples of human samples of normal oral mucosa and HNSCC (H&E stained). Note acetylated tumor cells (Ac. H3-FITC) that co-express high levels of vimentin (TRICT/green) at the invasion front of HNSCC. Epithelial cells in normal mucosa display acetylated cells distributed throughout the epidermis but do not express vimentin.

The *BMI-1* gene is upregulated in a variety of cancers and associated with increased tumor aggressiveness and poor survival rates [71], [92]–[97]. The oncogenic effect of BMI-1 is primarily mediated by suppressing the p16^ink4^ tumor suppressor gene, leading to activation of pRB and p53 signaling [98], [99]. We found that TSA-treated tumors showed increased expression of BMI-1 (Fig. 4A) localized in the nucleus (Fig. 4B), the development of a spindle shaped phenotype, and polarization of F-actin filaments (Fig. 4B). Interestingly, polarization of actin filaments was only observed in tumor cells treated with TSA. Normal human epithelial cells did not alter their morphology in response to chromatin decondensation, suggesting a selective effect of HDACi in tumor cells (Fig. S3_NOK-SI). Upregulation of BMI-1 was also associated with increased invasiveness of HN6 and HN13 cells (Fig. 4C, HN6 *p<0.05 and HN13 ***p<0.001). Collectively, we found that regardless of the original invasive capacity of HN6 and HN13 cells (Fig. 1B and C), chemical-induced chromatin acetylation caused HNSCC to undergo EMT (Fig. 3) and upregulate BMI-1 (Fig. 4A and B). Paradoxically, inhibition of HDACs also impaired the CSC population (Fig. 2C). These findings suggest that acetylation of HNSCC chromatin directly impairs the subpopulation of ALDH+ cancer cells, thereby selecting for a homogeneous subpopulation deprived of multipotency characteristics [16]–[25]. Furthermore, clinical use of HDACi is highly successful in treating hematologic malignancies that often behave as homogeneous diseases. Thus, epigenetic factors that modulate chromatin acetylation may be responsible for triggering changes in HNSCC behavior by alternating tumor cells between a quiescent and “stemness” stage that resists chemotherapy to a more aggressive and invasive behavior that promotes tumor metastasis. This mechanism is in line with the emerging tumorigenicity model of cellular “plasticity” and may represent an important mechanism used by HNSCC to simultaneously acquire an invasive behavior and chemoresistance. The initial use of HDACi may provide a molecularly defined window of opportunity for patients with head and neck cancer by chemically ablating tumor “plasticity” prior to the administration of genotoxic chemotherapy.

We further validated our findings in human samples of head and neck cancers and explored the acetylation status of tumor chromatin and the expression pattern of vimentin. Histone acetylation is considered a central switch that allows interconversion between permissive and repressive chromatin structures and domains [30]. Therefore, acetylation of core histones plays an important role in controlling gene expression by altering chromatin structure [100]. Surprisingly, we found that the invasion fronts of HNSCC tumors are characterized by increased chromatin acetylation. Additionally, vimentin co-localized with Ac.H3 in the invasion front of all analyzed tumors (Fig. 4D HNSCC and Fig. S4). Normal oral mucosa had a large number of cells staining for Ac.H3 but not vimentin (Fig. 4D_Normal Mucosa and Fig. S4), similar to what is observed in NOK-SI cells (Fig. 1A and 3A).

## Conclusions

**Figure 5:**
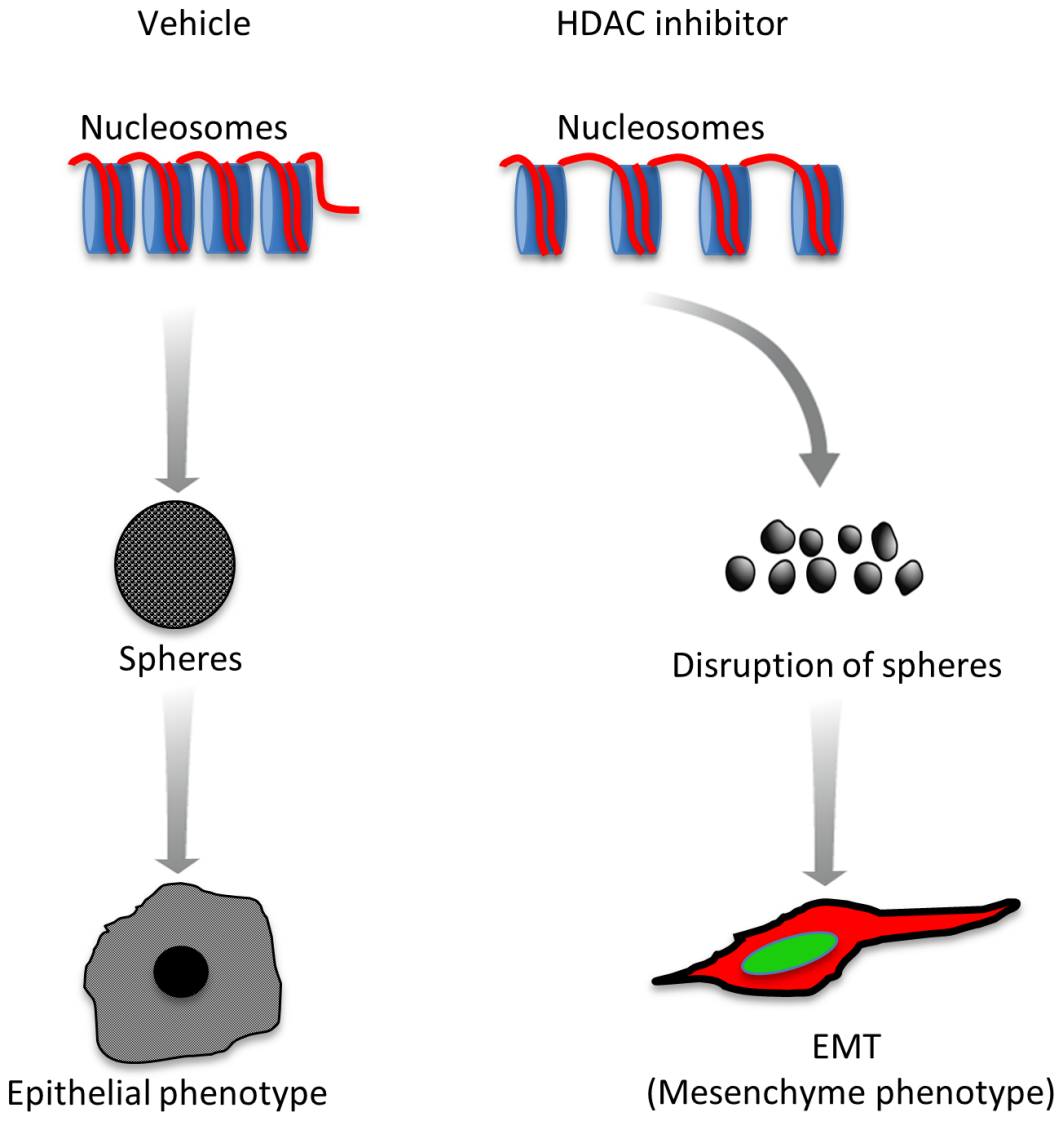
HDACi-induced EMT phenotype. Schematic drawing of the proposed mechanism by which chromatin acetylation influences head and neck tumor behavior. Tumors with hypoacetylated chromatin (low levels of Ac. H3) accumulate and maintain CSC. Pharmacological induction of chromatin acetylation (HDACi) reduces the CSC population and induces EMT, BMI-1 expression and aggressiveness of tumor cells.

Our results demonstrate that head and neck tumors are primarily hypoacetylated (low levels of Ac.H3), which may account for the accumulation and maintenance of CSC. We also observed that tumor cells respond differently to environmental cues by modulating chromatin acetylation and expression of invasion markers. Furthermore, pharmacological induction of chromatin acetylation induces aggressiveness, activation of EMT and increased expression of BMI-1 while simultaneously disrupting the CSC population (Fig. 5). The therapeutic implications of such findings are still under investigation. It is conceivable that disruption of CSC may directly affect tumor “plasticity”. Therefore, inhibition of HDAC may constitute a novel strategy to disrupt the population of CSC in head and neck tumors, thereby creating a homogeneous population of cancer cells with biologically defined signatures and predictable behavior.

## Supporting Information

Figure S1 **HNSCC invasion time course.** Invasion assay showing that 8 hrs is the optimal time needed for HNSCC to invade the fibronectin-coated polycarbonate filter membranes containing 8 µm-diameter pores. (A) Photomicrography of representative samples of human HNSCC cell lines (hematoxylin stained). (B) Graphic represents the percentage of cells invading the polycarbonate filter membrane in 4-hour intervals. At 12 and 16 hours, tumor cells have reached confluence.
(TIF)

Figure S2 **TSA dose response.** Western blot analysis showing acetylation of histone H3 Lys9 in HN13 cells. Expression of Ac. H3 was analyzed in response to TSA concentrations ranging from 100 to 600 nM. GAPDH served as a loading control.
(TIF)

Figure S3 **Representative images of immunofluorescence assays depicting the development of a spindle shaped phenotype and polarization of F-actin filaments (TRICT/red) following 24 hours of TSA (300 nM) treatment in NOK-SI and HNSCC cells.**
(TIF)

Figure S4 **Distribution of vimentin and BMI-1 proteins in human samples of normal oral mucosa and head and neck tumors.**
(TIF)
