# Supplementary figures and images for: Inhibition of Histone Deacetylase Impacts Cancer Stem Cells and Induces Epithelial-Mesenchyme Transition of Head and Neck Cancer
Source: PLoS One. 2013 Mar 20;8(3):e58672. doi: 10.1371/journal.pone.0058672 (PMC3603970; doi:10.1371/journal.pone.0058672)

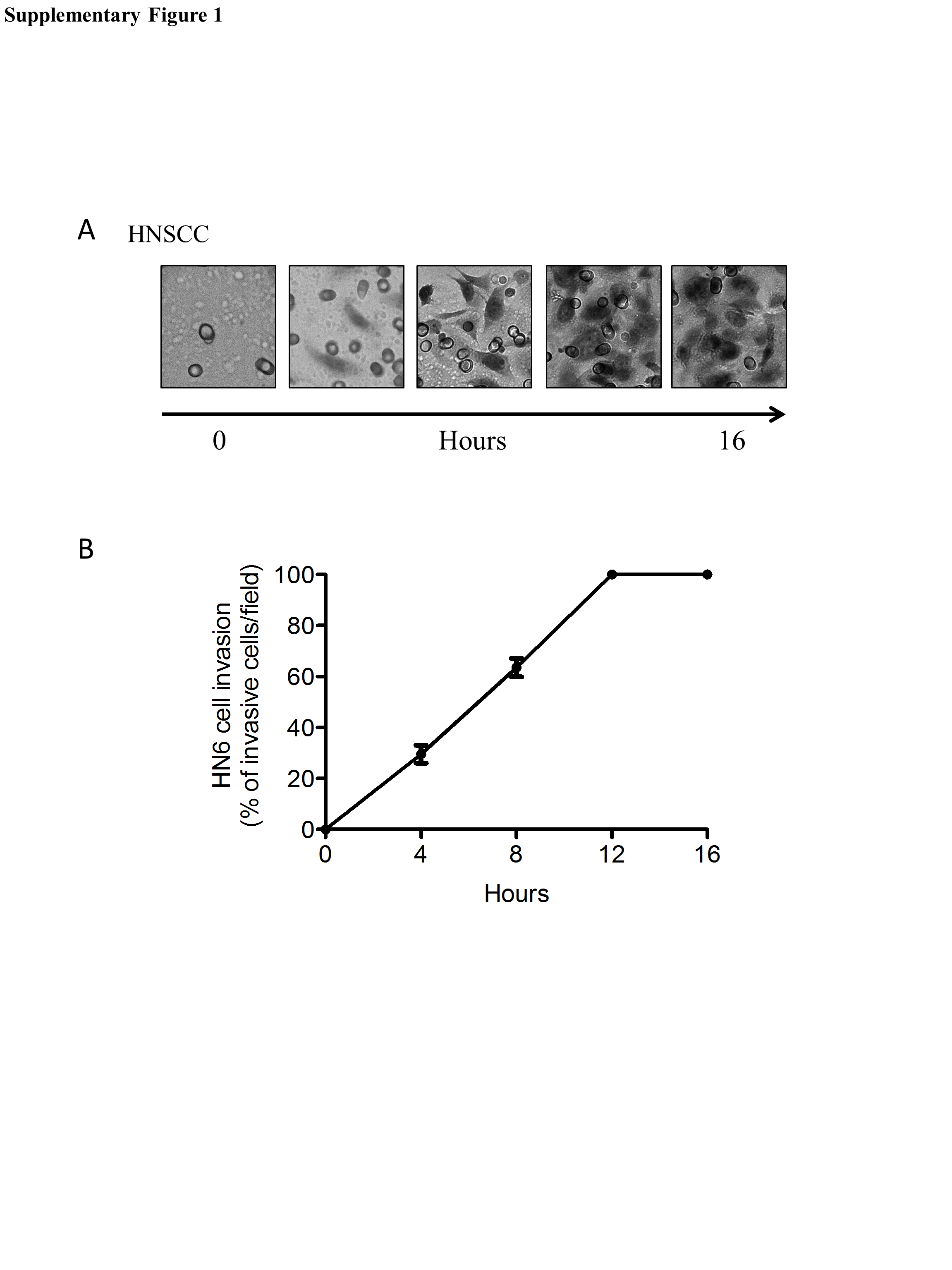

Supplement: Figure S1 — HNSCC invasion time course. Invasion assay showing that 8 hrs is the optimal time needed for HNSCC to invade the fibronectin-coated polycarbonate filter membranes containing 8 µm-diameter pores. (A) Photomicrography of representative samples of human HNSCC cell lines (hematoxylin stained). (B) Graphic represents the percentage of cells invading the polycarbonate filter membrane in 4-hour intervals. At 12 and 16 hours, tumor cells have reached confluence. (TIF) [file pone.0058672.s001.tif]

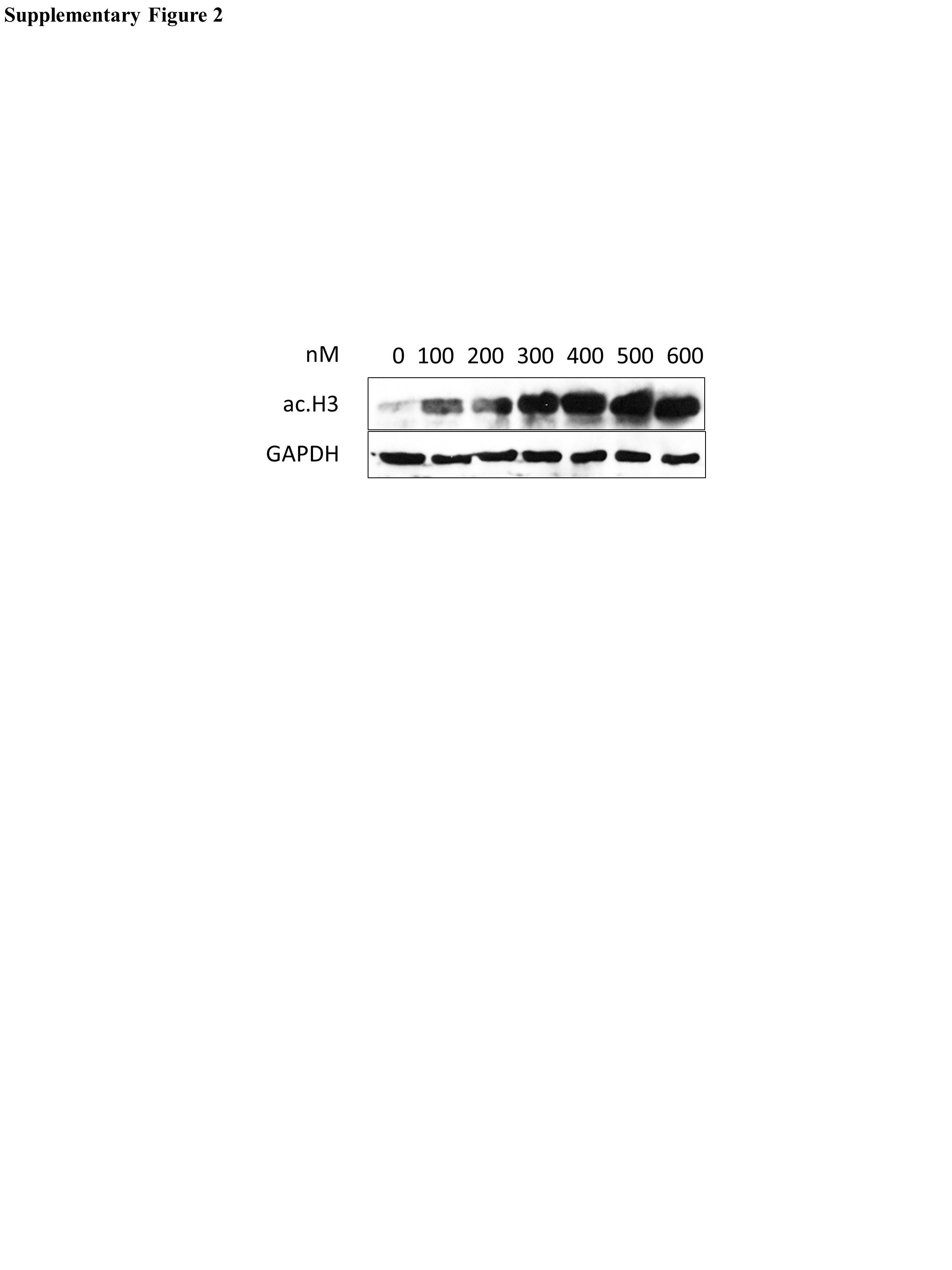

Supplement: Figure S2 — TSA dose response. Western blot analysis showing acetylation of histone H3 Lys9 in HN13 cells. Expression of Ac. H3 was analyzed in response to TSA concentrations ranging from 100 to 600 nM. GAPDH served as a loading control. (TIF) [file pone.0058672.s002.tif]

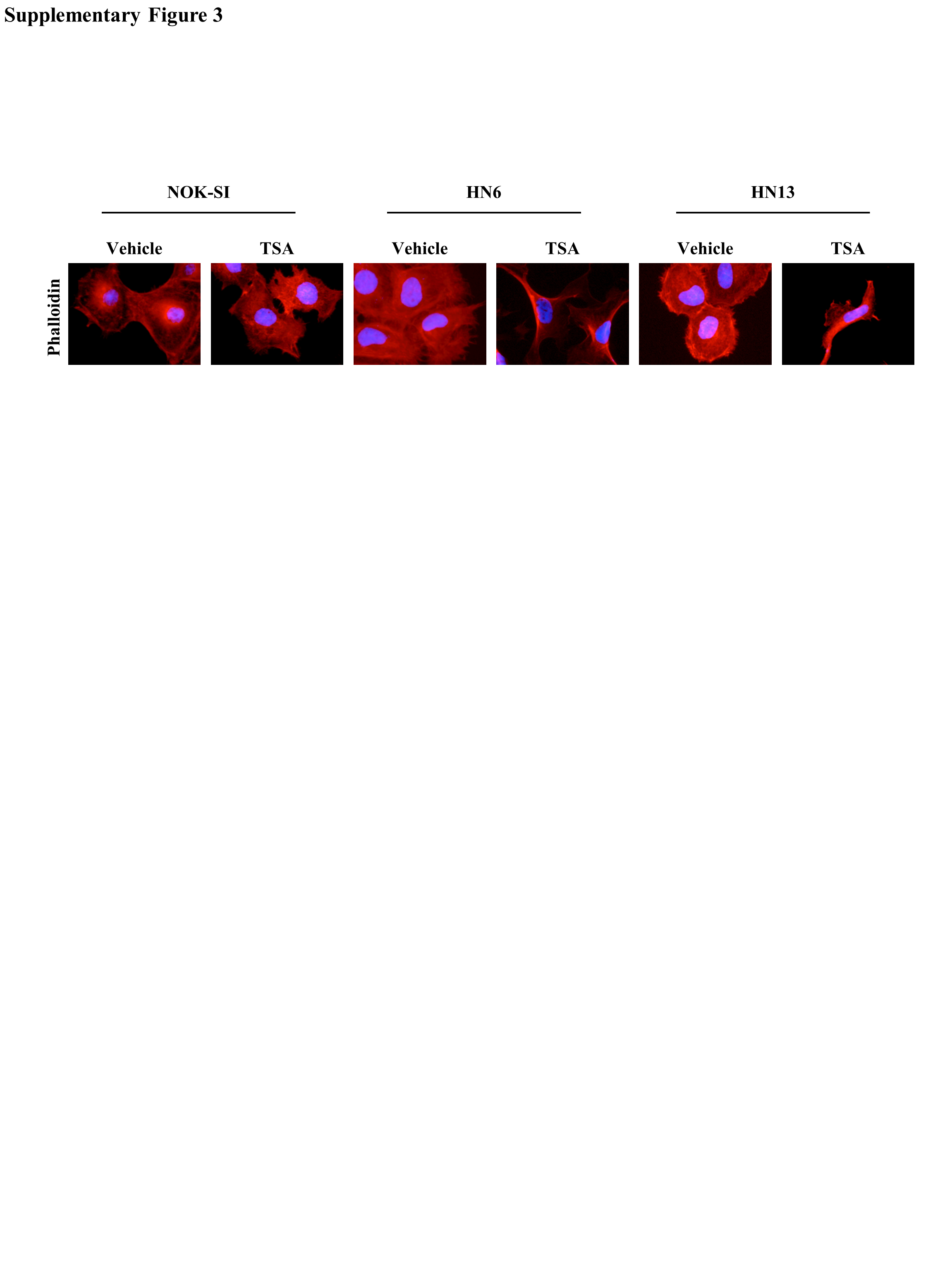

Supplement: Figure S3 — Representative images of immunofluorescence assays depicting the development of a spindle shaped phenotype and polarization of F-actin filaments (TRICT/red) following 24 hours of TSA (300 nM) treatment in NOK-SI and HNSCC cells. (TIF) [file pone.0058672.s003.tif]

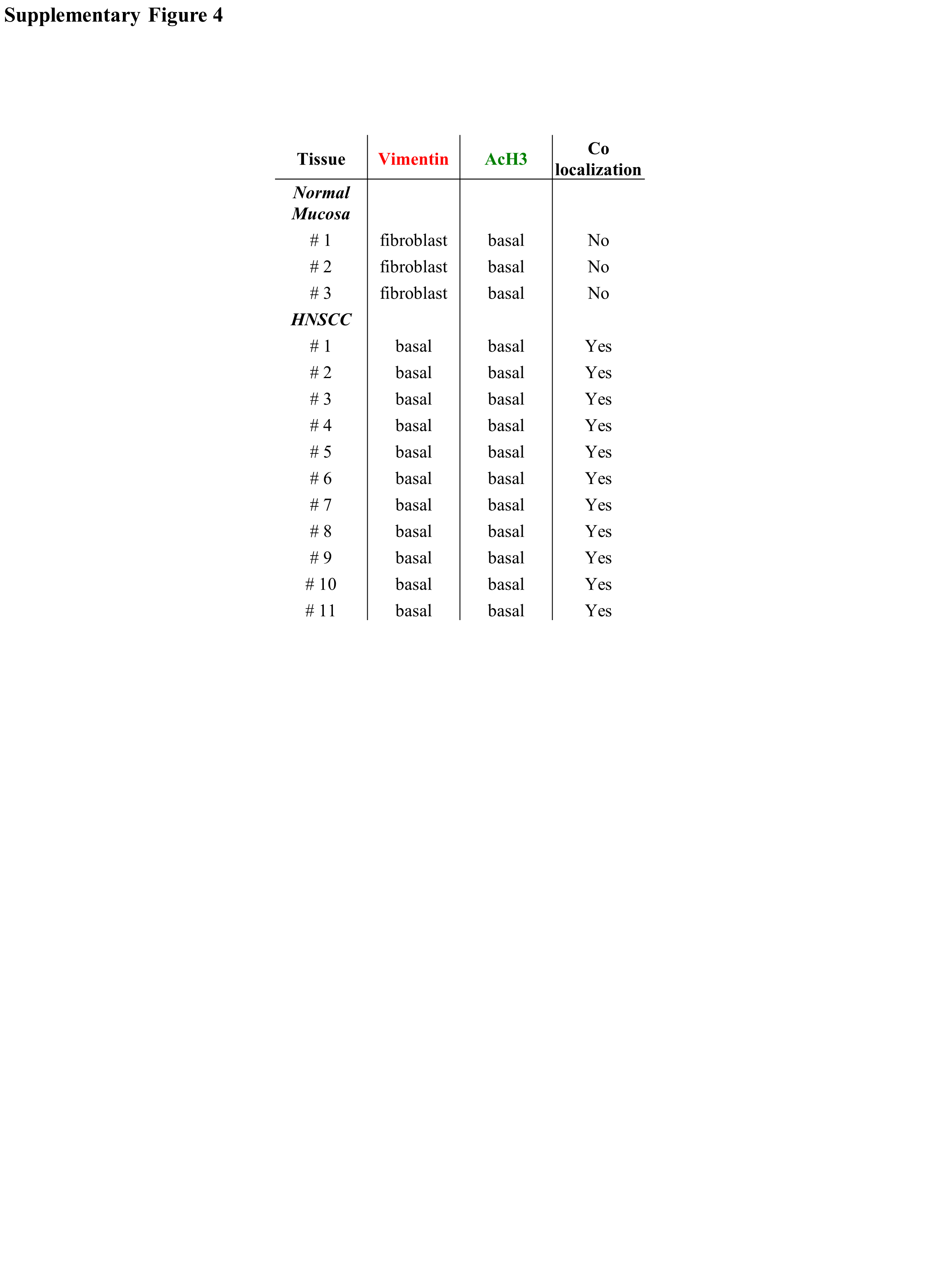

Supplement: Figure S4 — Distribution of vimentin and BMI-1 proteins in human samples of normal oral mucosa and head and neck tumors. (TIF) [file pone.0058672.s004.tif]
